# Depolymerization of actin filaments by Cucurbitacin I through binding G‐actin

**DOI:** 10.1002/fsn3.3804

**Published:** 2023-11-06

**Authors:** Ebru Haciosmanoglu Aldogan, Kemal Alper Önsü, Cemil Can Saylan, Başak Günçer, Sefer Baday, Muhammet Bektaş

**Affiliations:** ^1^ Department of Biophysics, Faculty of Medicine Bezmialem Vakif University Istanbul Turkey; ^2^ Department of Biophysics, Istanbul Faculty of Medicine Istanbul University Istanbul Turkey; ^3^ Chair of Experimental Bioinformatics, TUM School of Life Sciences Technical University of Munich Freising Germany; ^4^ Applied Informatics Department, Informatics Institute Istanbul Technical University Istanbul Turkey

**Keywords:** actin dynamics, cell migration, cucurbitacin I, F‐actin, G‐actin, target protein

## Abstract

Cucurbitacins have high economic value as they are a major source of food and have pharmacological properties. Cucurbitacin I (CuI) is a plant‐derived natural tetracyclic triterpenoid compound that shows an anticancer effect via inhibiting the JAK2‐STAT3 signaling pathway. The actin cytoskeleton is the most abundant protein in cells and regulates critical events through reorganization in cells. In this study, it is aimed at determining the direct effect of CuI on actin dynamics. The fluorescence profile of G‐actin in the presence of CuI (1–200 nM) shifted to a higher temperature, suggesting that G‐actin binds CuI and that G‐actin–CuI is more thermally stable than the ligand‐free form. CuI dose‐dependently inhibited the polymerization of F‐actin in vitro and disrupted actin filaments in endothelial cells. Docking and MD simulations suggested that CuI binds to the binding site formed by residues I136, I175, D154, and A138 that are at the interface of monomers in F‐actin. The migration ability of cells treated with CuI for 24 h was significantly lower than the control group (*p* < .001). This study reveals the molecular mechanisms of CuI in the regulation of actin dynamics by binding G‐actin. More importantly, this study indicates a novel role of CuI as an actin‐targeting drug by binding directly to G‐actin and may contribute to the mode of action of CuI on anticancer activities.

## Graphical abstract

Cucurbitacin I (CuI) is a plant‐derived natural tetracyclic triterpenoid compound that is one of the Cucurbitaceae family members. The study shows that CuI directly binds the actin cytoskeleton, and it could be a potent inhibitor of cell motility. Also, this study may contribute to the mode of action of CuI on anticancer activities.

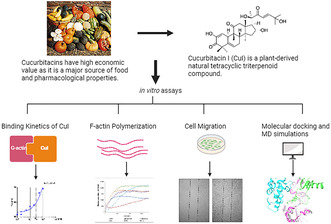

## 1 INTRODUCTION

Actin is the most abundant protein in the cell and plays a role in important tasks such as movement and division in the cell (Wehland et al., 1977). In addition, by interacting with regulatory proteins, it plays a role in intracellular signal transduction, intracellular transport, and the regulation of various enzyme activities (Hennessey et al., 1993). Actin is extremely valuable as a target for drug development because it may lead to changes in cellular vital activities, either directly or indirectly. Actin can be both in filamentous (F‐actin) and monomer (G‐actin) form in cells, and G‐actin is a form that is less studied for binding affinity studies. Cucurbitacins are triterpenoid natural compounds isolated from the Cucurbitaceae family of plants (Yang & Kim, 2018). It has been shown that cucurbitacins have anti‐inflammatory, anti‐cancer, and anti‐microbial effects with many signaling pathways, such as ERK1/2, JAK/STAT (Lee et al., 2010; Üremiş et al., 2022; Wu et al., 2016). Especially, cucurbitacin I (CuI) has an anticancer effect on several types of cancer cells, including lung cancer, glioma, and breast cancer, via inhibiting the JAK2/STAT3 pathway and suppressing the proliferation of cancer cells in vitro and in vivo *(*Alghasham, 2013; Chen et al., 2012; Kumar et al., 2023). Besides, it was shown that CuI inhibits STAT3 but induces STAT1 through disturbing actin filaments in cancer cell lines (Guo et al., 2018). However, the molecular mechanisms of its action and the relationships between the anti‐cancer effect and disturbing actin filaments have not yet been elucidated. In this study, we investigated the direct interaction of CuI and monomeric actin (G‐actin), the effect of CuI on F‐actin polymerization, and the interaction side between CuI and actin. Here, we report that CuI disturbs actin filaments with binding G‐actin and inhibits actin polymerization in vitro and in cells. These results indicate that CuI may show its inhibitory effect on cancer cell proliferation, invasion, and migration by disrupting F‐actin through its interaction with G‐actin, thereby impeding these cellular processes.

## 2 MATERIALS AND METHODS

### 2.1 Differential scanning fluorimetry

Rabbit skeletal muscle G‐actin (1 mg/mL) (Cytoskeleton, Inc., AKL99, Denver, CO, USA), SYPRO orange at 50× (stock: 5000×, Thermo Fisher Scientific, Waltham, MA, USA), and increasing concentrations (1, 2, 5, 10, 25, 50, 100, and 200 nM) of CuI (Sigma, St. Louis, MO, USA) were incubated in a PCR microplate and heated from low‐to‐high temperature (25 to 90°C) at a slow rate (0.5°C/15 s) in a real‐time PCR machine (BioRad CFX 96, California, USA). All samples were studied in triplicate, and the denaturation (melting) midpoint (*T*_m_) was determined by plotting the first derivative of fluorescence versus temperature and finding the temperature at the midpoint of the transition with the Boltzmann equation. The data analysis was performed using CFX Manager and GraphPad software, and protein–ligand dissociation constants (*K*_D_) were calculated (Gedgaudas et al., 2022; Huynh & Partch, 2015). Graphs were presented as normalized plots in Excel.

### 2.2 Pyrene‐actin polymerization assay

Rabbit skeletal muscle actin from Cytoskeleton, Inc. was labeled with pyrene as previously described (Carlier et al., 1984). Ca‐ATP‐actin was converted into Mg‐ATP‐actin in G‐buffer (5 mM Tris, 0.2 mM ATP, 0.1 mM CaCl_2_, 1 mM DTT, 1 mM MgCl_2_, and 0.2 mM EGTA, pH:7.6) and incubated on ice for 2 h. Actin polymerization was monitored in (5 mM Tris, 50 mM KCl, 0.2 mM ATP, 0.1 mM CaCl_2_, 1 mM DTT, 1 mM MgCl_2_, 0.2 mM EGTA, pH:7.6) containing increasing CuI concentrations compared with measurements of jasplakinolide (15 μM) and cytochalasin D (15 μM) (Sörensen et al., 2012). The increase in fluorescence of 10% pyrenyl‐labeled actin was monitored using a fluorescence spectrophotometer (λexc = 365 nm, λem = 407 nm). All measurements were repeated at least three times (Romero et al., 2007).

### 2.3 Cell culture

Human umbilical vein endothelial cells (HUVECs) were obtained from the American Type Culture Collection (ATCC® CRL‐1730, Manassas, VA, USA) and cultured in DMEM‐F12 medium supplemented with 10% fetal bovine serum and 1% penicillin and streptomycin at 37°C in 5% CO_2_. Cells were seeded into a 6‐well plate (10^5^ cells/well) and incubated overnight prior to treatment with increasing concentrations of CuI (50, 100, 200, and 400 nM) for 24 h (Capes‐Davis et al., 2021).

### 2.4 Fluorescence microscopy

HUVECs were permeabilized with 0.5% Triton X‐100 and then fixed in 2% paraformaldehyde in PBS. After blocking with 2% BSA, cells were incubated with Phalloidin‐FITC (1 μg/mL) for visualization of total actin. All images were acquired with an Olympus BX51 Research Microscope and DP72 camera controlled by Olympus DP2‐TWAIN software (Bektas et al., 2011).

### 2.5 Cell migration assay

HUVECs (1 × 10^6^) were seeded into a 6‐well plate and grown to confluence for the cell migration assay. Wounds in the cell monolayer were obtained by scratching with a pipet tip. Detached cells were washed, and new medium was added with or without CuI. Cells were photographed with a Zeiss Primovert microscope and an Axiocam ICC5 camera at 0 and 24 h, and the area of each scratch was measured with the ImageJ program with the Wound Healing Tool Patch (Altinkaynak et al., 2023).

### 2.6 Molecular docking and dynamic simulations

The blind docking method was used to identify potential CuI (ID: ZINC000004097803) binding sites on G‐actin (PDB ID: 3HBT). Two different docking software programs (Vina (Trott & Olson, 2010) and LeDock (Wang et al., 2016)) were used. The grid box was set to cover complete protein. Since docking calculations are stochastic calculations, seven docking runs were carried out with different seed numbers. The candidate binding pose scores were compared against 500 random ligand binding scores. The 500 ligands were selected based on their similarity with CuI using DataWarriar. Molecular dynamics simulations were conducted using Desmond (Bowers et al., 2006). TIP3P water was used to solvate the ligand–receptor complexes within a cubic box that was 10 Å away from the receptor surface. The system was neutralized with a counterion, and a 0.15 M NaCl salt solution was added. Maestro 11.8 was used to prepare the systems, and the OPLS 2005 force field was used (Jorgensen et al., 1996). The particle mesh Ewald method was used to calculate electrostatic interactions, while van der Waals and coulombic interactions were defined with a radius of 9 Å cut‐off. During production, the temperature was kept at 310 K with the Nosé–Hoover thermostat, and the system pressure was maintained at 1.01325 bar using the Martyna–Tobias–Klein method. Additionally, we used the SHAKE algorithm and set the time step to 2.0 fs. Before production, we applied a default NPT ensemble relaxation protocol that was available at Maestro relaxation protocol examples. The production simulations were performed for 100 ns.

### 2.7 Statistical analysis

Data are expressed as mean ± standard error of mean (SEM). One‐way ANOVA (analysis of variance) with post hoc comparisons based on Tukey's multiple comparisons test was applied. Differences were considered significant at an error probability of *p* < .05. GraphPad Prism 5.0 (Graphpad Software, La Jolla, CA) was used for all statistical analyses.

## 3 RESULTS

### 3.1 Effect of CuI on the shift of protein melting point (*T*_m_)

**FIGURE 1:**
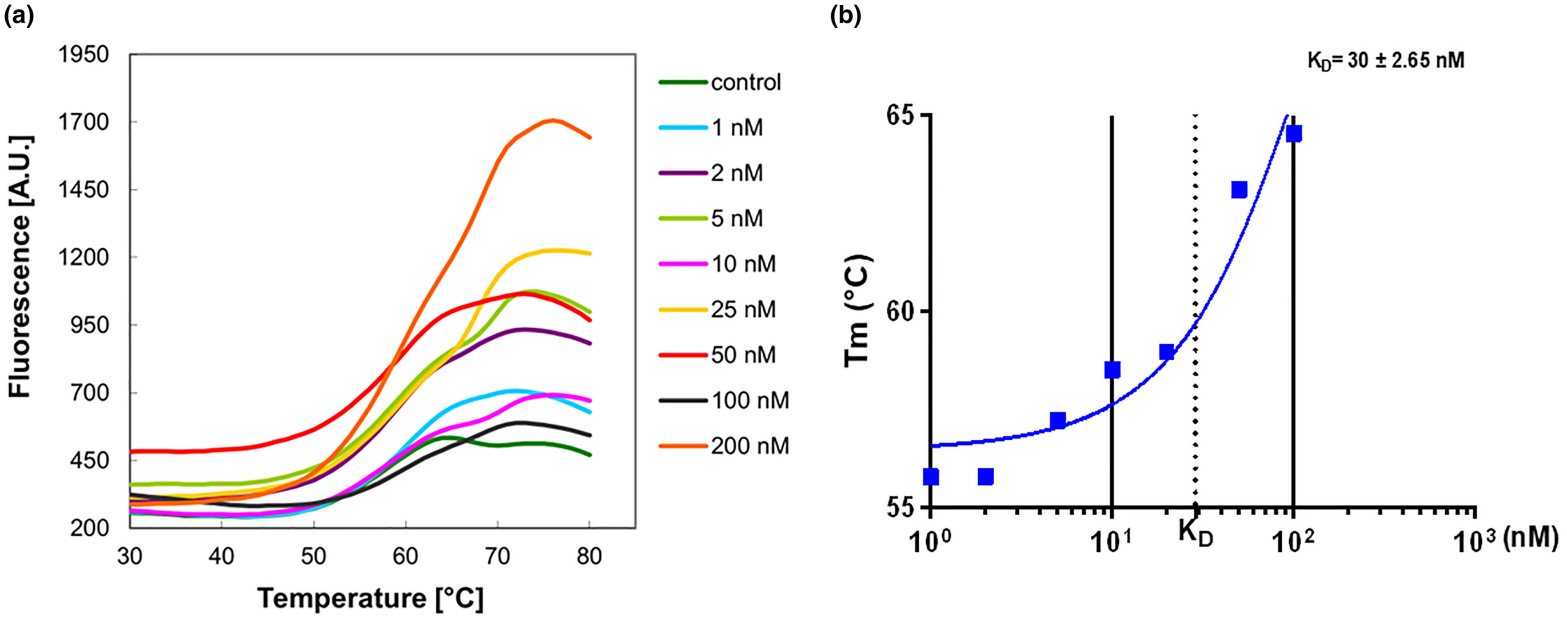
In vitro characterization of CuI binding to G‐actin. (a) DSF of G‐actin was incubated with CuI in different concentrations. G‐actin alone is in green, 1 nM in turquois, 2 nM in purple, 5 nM in light green, 10 nM in pink, 25 nM in yellow, 50 nM in red, 100 nM black, and 200 nM in orange. (b) The binding of CuI to G‐actin is calculated by DSF. The calculated *K*_D_ value is 30 μM.

Differential scanning fluorimetry (DSF) measurements were realized to describe the effect of CuI on the thermodynamic stability of G‐actin. G‐actin shows a melting temperature (*T*_m_) of approximately 56.51°C. Meanwhile, upon incubation with CuI (200 nM), this value increases to 63.63°C. A significant shift in the protein's *T*_m_ of 8.2°C (∆*T*_m_) is observed in the presence of the ligand (Figure 1a, Table 1). The calculated *K*_D_ for the G‐actin–CuI complex is 30 ± 2.5 nM (Figure 1b) (Vivoli et al., 2014). This drastic change in protein resistance to thermal denaturation indicates a strong stabilizing interaction between CuI and G‐actin. Besides, G‐actin can directly interact with CuI.

**TABLE 1:** *T*_m_ and the difference (∆*T*_m_) in *T*_m_ in the absence and presence of CuI.

Concentration of CuI (nM)	*T*_m_ (°C)	*T*_m_ slope (∆_Tm_, °C)
Control	56.51	3.2
1	57.65	4.2
2	58.65	4.9
5	59.11	6.1
10	59.81	5.4
25	61.06	7.2
50	61.1	6.3
100	62.19	6.2
200	63.63	8.2

### 3.2 Effect of CuI on actin polymerization and cytoskeletal organization

**FIGURE 2:**
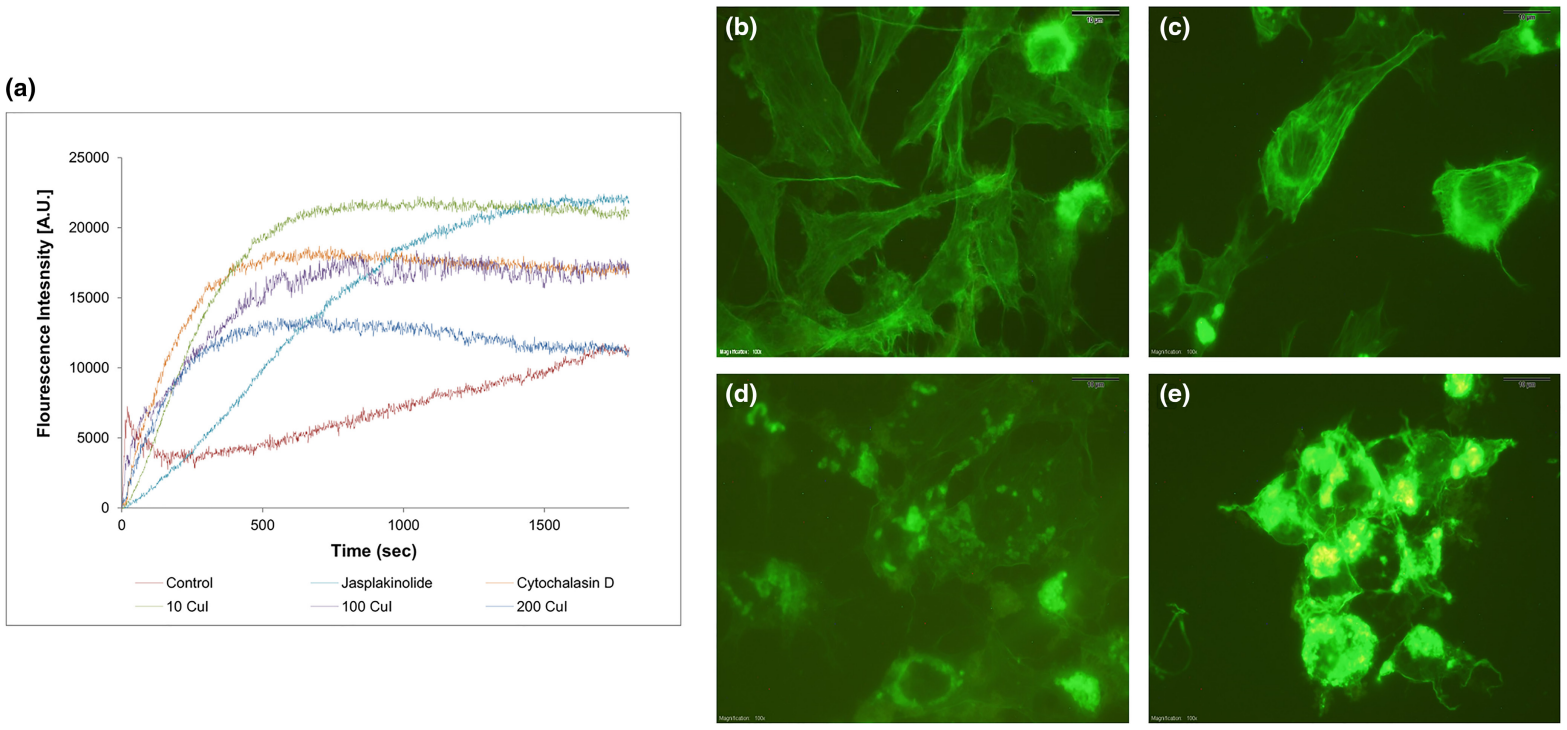
Effect of CuI on actin polymerization. (a) In vitro actin‐pyrene polymerization assay with CuI (10–100‐200 nM), jasplakinolide (15 μM), cytochalasin D (15 μM), and control. G‐actin (20 μg/mL) spiked with 10% pyrene‐labeled actin was incubated with small molecules. Effect of CuI on the actin cytoskeleton of HUVECs after CuI treatment. Cells were stained with phalloidin‐FITC (green) for F‐actin. (b) Control (no treatment); (c) 10 nM CuI; (d) 100 nM CuI; (e) 200 nM CuI; Scale bars, 10 μm.

To investigate the impact of CuI on F‐actin polymerization, we performed the kinetics of fluorescence enhancement from 0 to 60 min (Figure 2a). Polymerization rates were calculated from the slopes of the linear regions of each plot during the filament growth phase to compare with jasplakinolide as an actin stabilizer and cytochalasin D as decreasing polymer formation. Although 10 nM CuI had only a minimal effect on the rate of actin polymerization (*V*_50_ = 436.1 ± 2133), it has been observed that 200 nM CuI inhibits actin polymerization rate ~10‐fold in vitro (*V*_50_ = 104.1 ± 10.45) according to control (*V*_50_ = 1149 ± 11.63) (Table 2). To determine if CuI in vitro binding is relevant in vivo, F‐actin fibers were visualized in HUVECs with Phalloidin‐FITC by fluorescence microscopy (Figure 2b). It was observed that CuI induced a disturbing actin cytoskeleton at a 100 nM concentration with the destruction of F‐actin distribution (Figure 2c–e). As confirmed by fluorescence microscopy, both in vivo and in vitro experiments indicated that CuI disturbs F‐actin with CuI–G‐actin complex by direct interaction.

**TABLE 2:** Effect of CuI on actin polymerization rate (*V*_50_) in vitro (rates were calculated from slopes of the linear segment of curves corresponding to half maximal polymerization).

	*V*_50_
Control	1149 ± 11.63
Jasplakinolide (15 μM)	54.7 ± 5114
Cytochalasin D (15 μM)	77.59 ± 7776
10 nM CuI	436.1 ± 2133
100 nM CuI	154.7 ± 2016
200 nM CuI	104.1 ± 10.45

### 3.3 Inhibitory effect of CuI on cell migration

**FIGURE 3:**
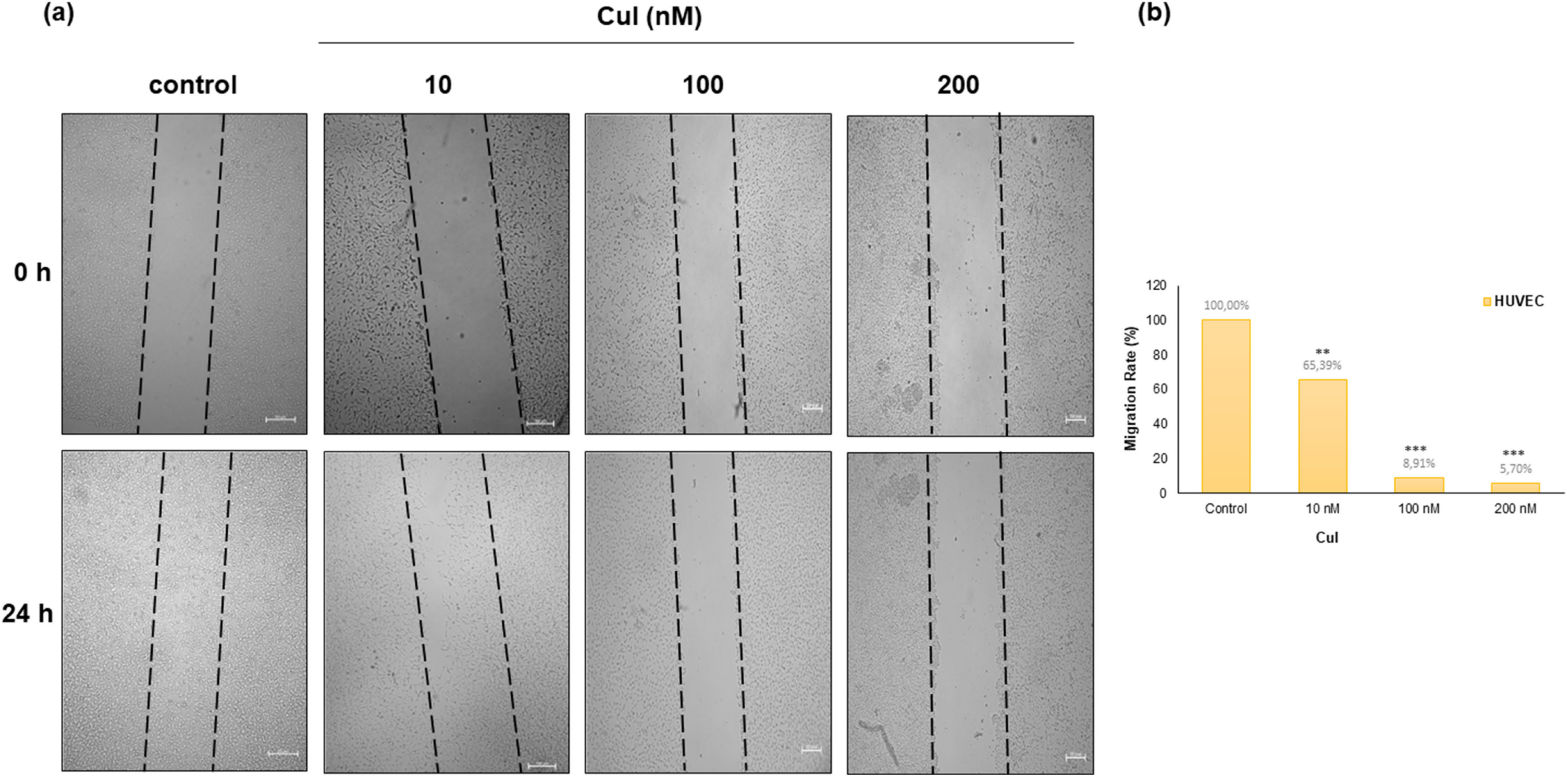
Detection of cell migration ability after treatment with CuI (10–100‐200 nM) using a wound healing assay. (a) Cells were wounded by scratching with a pipette tip and were incubated for 24 h after treatments. The cells were photographed under phase‐contrast microscopy (×4 magnificaion). (b) Migration was calculated with respect to the control conditions in the lineal phase (* *p* < .05, **p < .01, ***p < .001) by imageJ program with the Wound Heal Tool Patch.

To verify the properties of CuI on actin‐based disruptions involved in cell migration, an in vitro cell migration scratch assay was carried out. It is important to mention that cell migration is driven by the polymerization/depolymerization of actin (Bonfim et al., 2021). (Figure 3a). The migration assay revealed a 34.61% reduction in the number of migratory cells of 10 nM CuI group compared with the control group within 24 h. Besides, a scratch test showed a 94.30% decrease in migration ability at 24 h in cells subjected to 200 nM CuI compared with control cells (Figure 3b). The result suggested that HUVECs' migration was significantly inhibited by CuI.

### 3.4 Molecular docking and MD simulations

**FIGURE 4:**
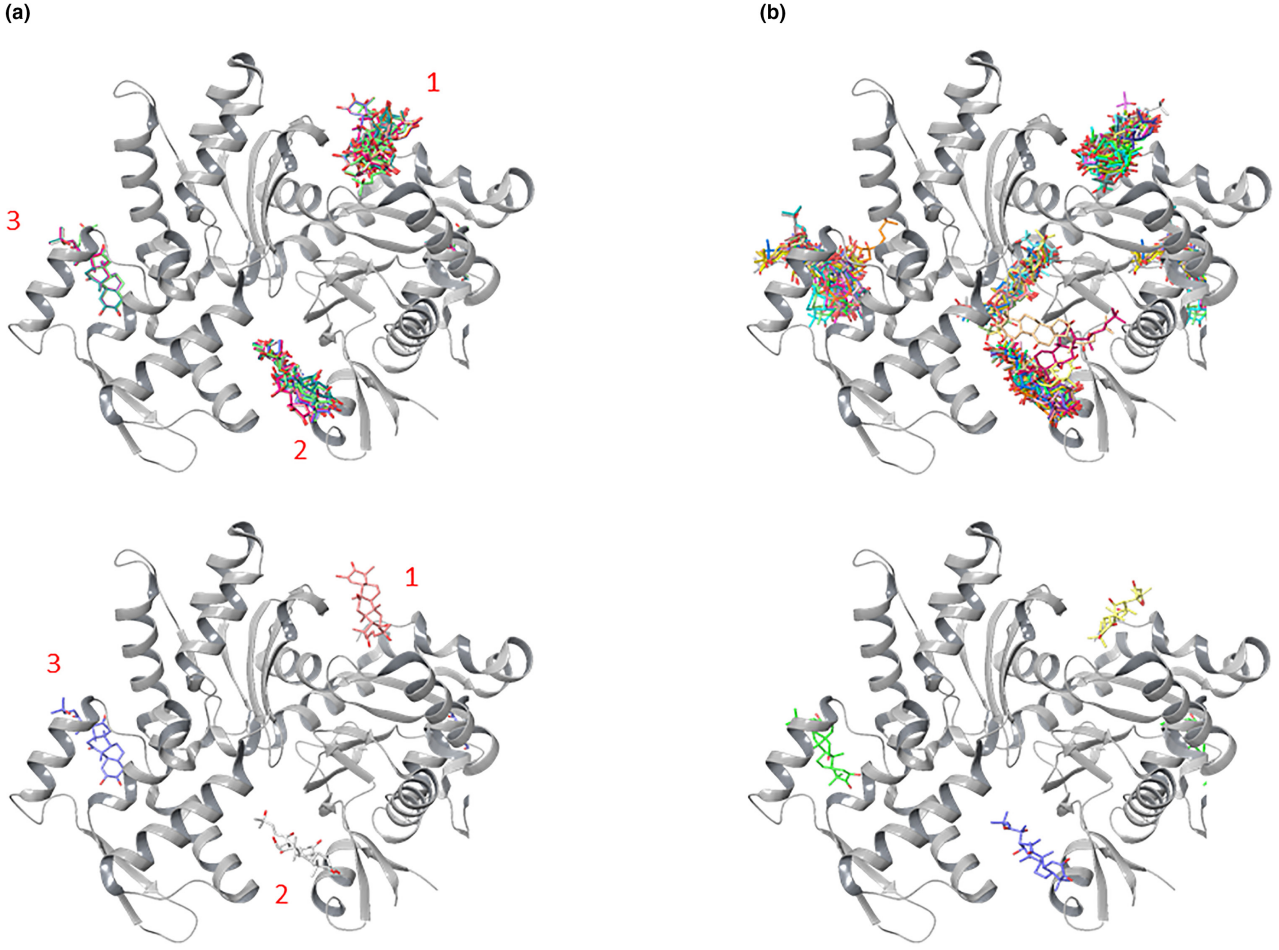
Potential CuI binding sites on the G‐actin using the Vina (a) and LeDock (b) programs. Figures on the lower panel display the highest scoring binding pose for each potential site. The numbers represent the order according to the docking scores.

We predicted potential binding sites for CuI on G‐actin using the Vina and LeDock docking programs. Seven different docking runs were performed in order to see where the protein–ligand binding poses are populated. Three particular binding sites showed the highest distribution of ligand binding, with the best scores being (Gibbs free energy −∆*G*) −8.3, −8.1, and −7.7 kcal/mol, respectively (Figure 4a). The dissociation constant *K*_D_ is related to Gibbs free energy ∆*G* by the relation ∆*G* = −R T ln (*K*_D_). Similarly, LeDock showed three binding sites with scores of −6.31, −5.61, and −5.37 kcal/mol, respectively (Figure 4b). Overall, the Vina and LeDock programs agree on the potential binding sites.

The docking score itself is not a strong identifier that can be used to explain if a ligand binds to a protein or not. Thus, in order to estimate whether CuI has a significant binding or behaves like a random ligand, we performed docking experiments using Vina and LeDock with 500 random ligands obtained from the ZINC Diverse Database. The CuI showed higher binding scores for all three binding sites against random ligands, which suggest a specific binding relationship for these sites (Figure S1).

**FIGURE 5:**
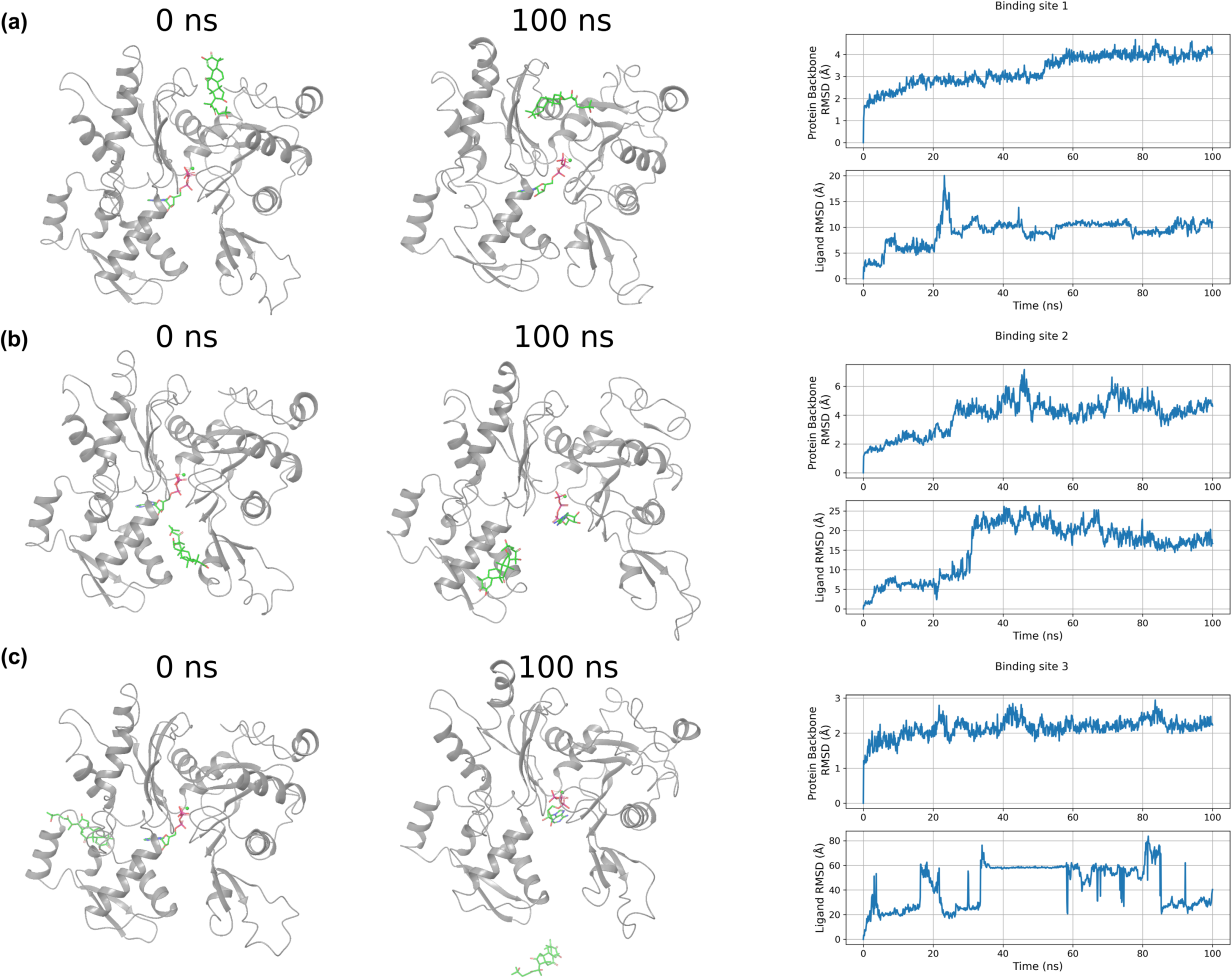
MD simulations of CuI in complex with G‐actin. Panels (a), (b), and (c) display results for the simulations started with CuI placed at binding sites1, 2, and 3, respectively. For each simulation, snapshots at the beginning and at the end of 100 ns are shown.

Three MD simulations of G‐actin in a complex with CuI were performed to investigate the stability of CuI at each potential binding site. The CuI at binding site 1 exhibited the most stable trajectory compared with the CuI placed at binding sites 2 and 3 (Figure 5a–c). CuI placed at binding site 3 flew away from the binding site. CuI placed at the binding site 1 has lower RMSD values compared with the trajectory of CuI placed at the binding site 2, suggesting CuI is more stable at binding site 1.

**FIGURE 6:**
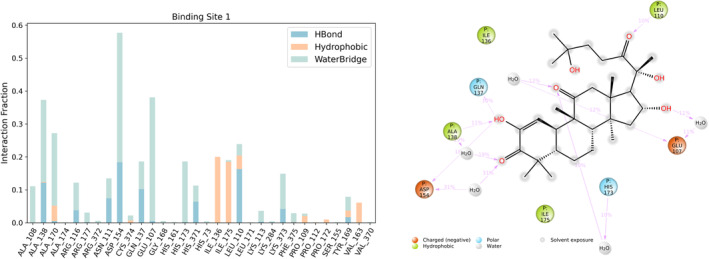
Interaction diagram for CuI and G‐actin for binding site 1.

Interaction analyses obtained from the simulation trajectory of CuI with G‐actin at binding site 1 Figure 6. Important interactions between CuI and G‐actin are explained as follows: Glu107, Gln137, and Leu110 (backbone) residues form hydrogen bonds with CuI. Asp154, His173, and Ala138 (backbone) residues are interacting with CuI through a water bridge. Ile175 and Ile136 make hydrophobic contacts with CuI.

**FIGURE 7:**
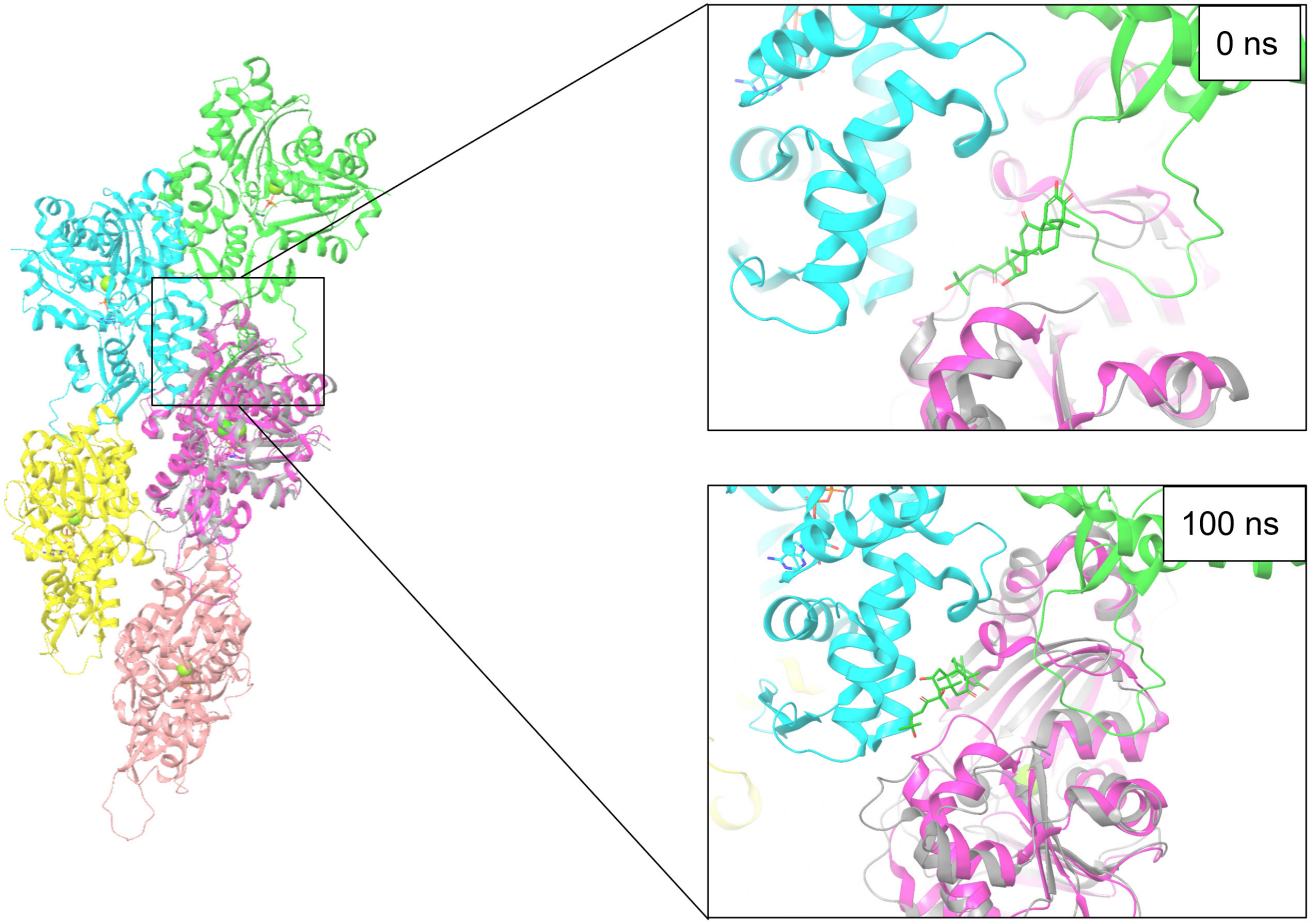
Displaying CuI binding site 1 on the F‐Actin structure.

The CuI binding for 0 and 100 ns at binding site 1 for F‐actin was manually assessed by aligning the G‐actin structures. Since the location of site 1 is the intersection with other actin monomers in the F‐actin structure, we suggest that CuI may have the potential to disrupt F‐actin formation through this binding site (Figure 7). Moreover, the fact that CuI is close to Cys374 at binding site 1 brings speculation for covalent binding, which was shown to be the case for CuE (Figure S2) (Sörensen et al., 2012).

## 4 DISCUSSION

Family Cucurbitaceae has been used for broad applicability in pharmacological activities like anti‐cancer, anti‐inflammatory, and cardiovascular protection since ancient times. Although many studies have shown that cucurbitacins, especially CuI, inhibit the STAT3 pathway and disrupt actin filaments, their action mechanism is still controversial (Guo et al., 2018; Patel & Ghane, 2021; Zhang et al., 2011). The actin cytoskeleton plays important roles in most cellular processes, including mechanical support, cell migration, differentiation, and signaling pathways. F‐actin contributes to morphology, trafficking, migration, and adhesion through continuous polymerization and depolymerization (Alvarez‐Rivera et al., 2023). Cucurbitacins generally affect the organization of the actin cytoskeleton, causing changes in the cells' normal actin dynamics and actin aggregate formation. Although it has been reported that cucurbitacin I inhibits cell motility by indirectly interfering with actin dynamics (Knecht et al., 2010), we have observed that CuI can be a potent inhibitor of cell motility by direct binding. We analyzed the binding constant of CuI to G‐actin by differential scanning fluorimetry, and an 8.2°C thermal shift was observed with CuI treatment (200 nM) according to G‐actin alone. Binding affinity was examined and found to be 30 ± 2.5 nM for the G‐actin–CuI complex, and it is known that a lower *K*_D_ value (lower concentration) is correlated with a higher affinity for the protein (Salahudeen & Nishtala, 2017). Besides, the F‐actin polymerization rate was analyzed and compared with the other actin‐binding drugs, jasplakinolide and cytochalasin D, and it has been realized that CuI (200 nM) reduced the polymerization rate of F‐actin nearly as much as jasplakinolide and cytochalasin D. In our study, the formation of large cytoplasmic actin aggregates was observed in HUVECs by fluorescence microscopy, similar to Kenecht's study (Knecht et al., 2010). Recent studies have shown that CuB has anti‐migration ability in cancer cell lines (Huifu et al., 2023; Kaewmeesri et al., 2022). Although anti‐migration activity seems to be due to inhibition of STAT3 and enhancement of STAT1 signaling, our results suggest that the reason could be binding G‐actin (Guo et al., 2018).

**FIGURE 8:**
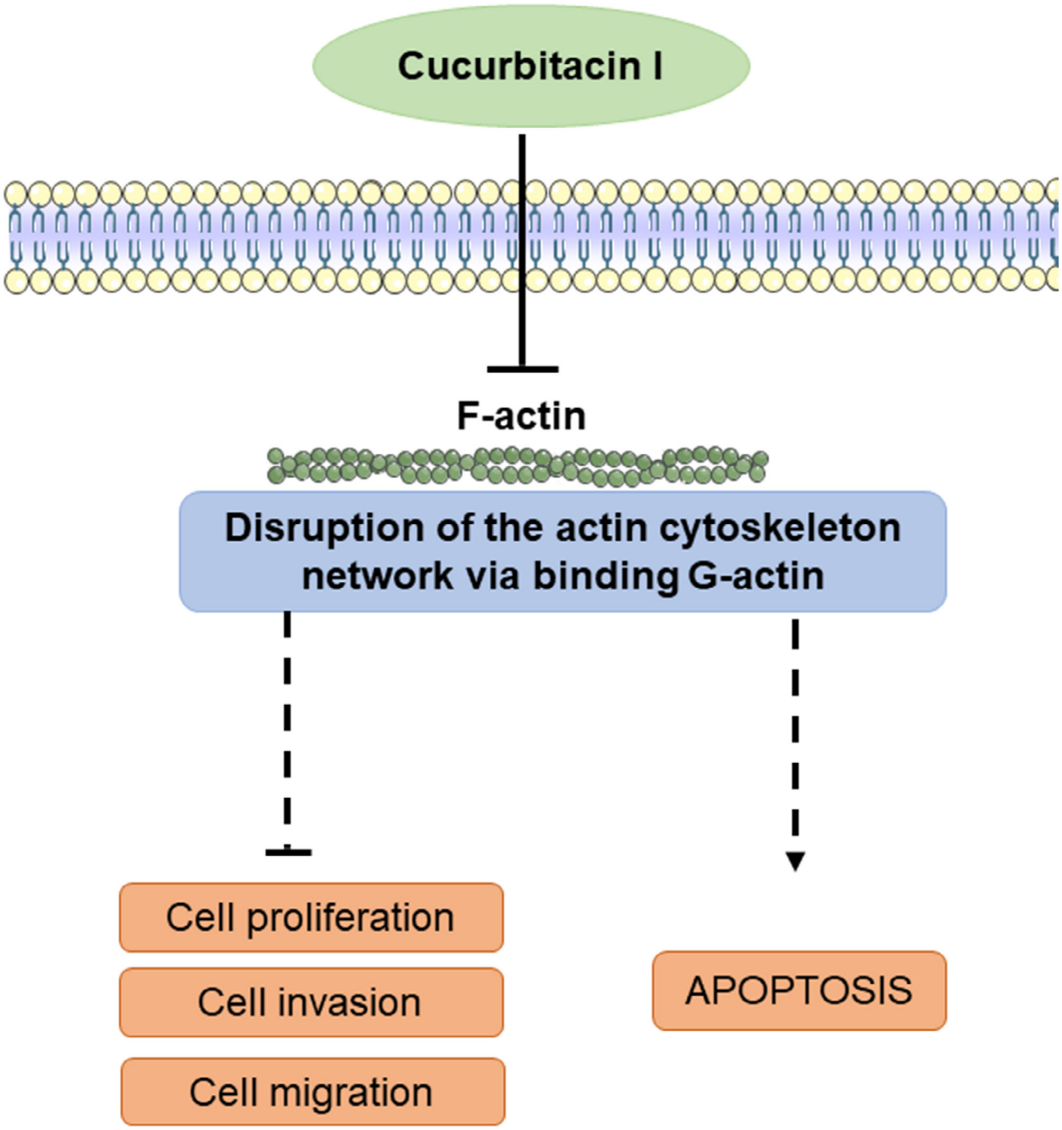
Schematic representation of possible mechanisms of action of CuI (↑: upregulation, ┴: downregulation) (Delgado‐Tiburcio et al., 2022; Kumar et al., 2023).

Interestingly, one of the cucurbitacins, CuE, modulates the actin cytoskeleton by forming an irreversible covalent bond with Cys257 of actin, and it has been reported that it is closely situated to the ATP‐binding site according to docking studies (Kumar et al., 2016; Roopa et al., 2020). In our findings, CuI showed higher binding scores for all three binding sites on G‐actin, specifically. Besides, CuI is close to bind on Cys374 at binding site 1 brings speculation for covalent binding on F‐actin that CuI may have the potential to disrupt F‐actin formation through this binding site. Based on our findings, CuI may realize its mode of action with binding G‐actin. Defining the role of the CuI–G‐actin interaction could critically inform the underlying anticancer mechanism (Figure 8).

In summary, our results indicated that CuI reduces F‐actin polymerization and cell migration via binding G‐actin. CuI could be a potent inhibitor of cell motility and an actin‐binding drug that disrupts actin dynamics, which may be useful to understand the biological activities of CuI on cancer cells. On the other hand, the G‐actin–CuI interaction side should be clarified for further experiments to define the proposed function. Besides, G‐actin–CuI interactions need further investigation to evaluate their relations with anticancer mechanisms.

## AUTHOR CONTRIBUTIONS

**Ebru Haciosmanoglu Aldogan:** Data curation (equal); investigation (equal); methodology (lead); validation (equal); visualization (equal); writing – original draft (lead); writing – review and editing (lead). **Kemal Alper Önsü:** Data curation (equal); methodology (supporting); resources (supporting). **Cemil Can Saylan:** Data curation (equal); investigation (equal); methodology (equal); validation (equal); visualization (equal); writing – original draft (equal); writing – review and editing (equal). **Başak Günçer:** Conceptualization (equal); methodology (equal); project administration (equal); supervision (equal); writing – original draft (equal). **Sefer Baday:** Conceptualization (equal); data curation (equal); methodology (equal); software (equal); supervision (equal); validation (equal); visualization (equal). **Muhammet Bektaş:** Conceptualization (equal); investigation (equal); project administration (equal); resources (equal); supervision (lead); writing – original draft (equal); writing – review and editing (equal).

## FUNDING INFORMATION

This work was supported by TUBITAK/2214‐A (Project Number: 1059B141400326) and the Scientific Research Foundation of Istanbul University (Project Number: 30522).

## CONFLICT OF INTEREST STATEMENT

The authors declare no conflict of interest.

## Supporting information


Figures S1–S2

## Data Availability

The data that support the findings of this study are available on request from the corresponding author. The data are not publicly available due to privacy or ethical restrictions.
