# Supplementary material for: Depolymerization of actin filaments by Cucurbitacin I through binding G‐actin
Source: Food Sci Nutr. 2023 Nov 6;12(2):881–9. doi: 10.1002/fsn3.3804 (PMC10867458; doi:10.1002/fsn3.3804)
Supplement: Supplementary file 1 — Figures S1–S2 [file FSN3-12-881-s001.docx]

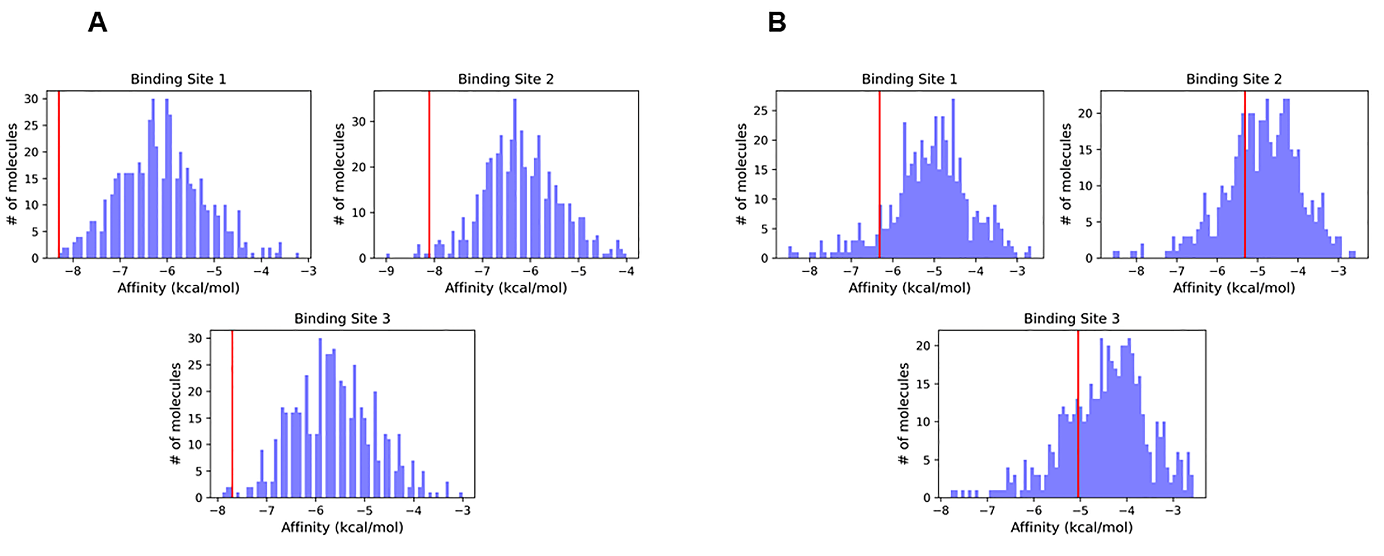


**Figure S.1.** Distribution of docking scores of 500 random ligands docked via Vina **(A)** and LeDock **(B)**. Red straight lines show the binding score for CuI.


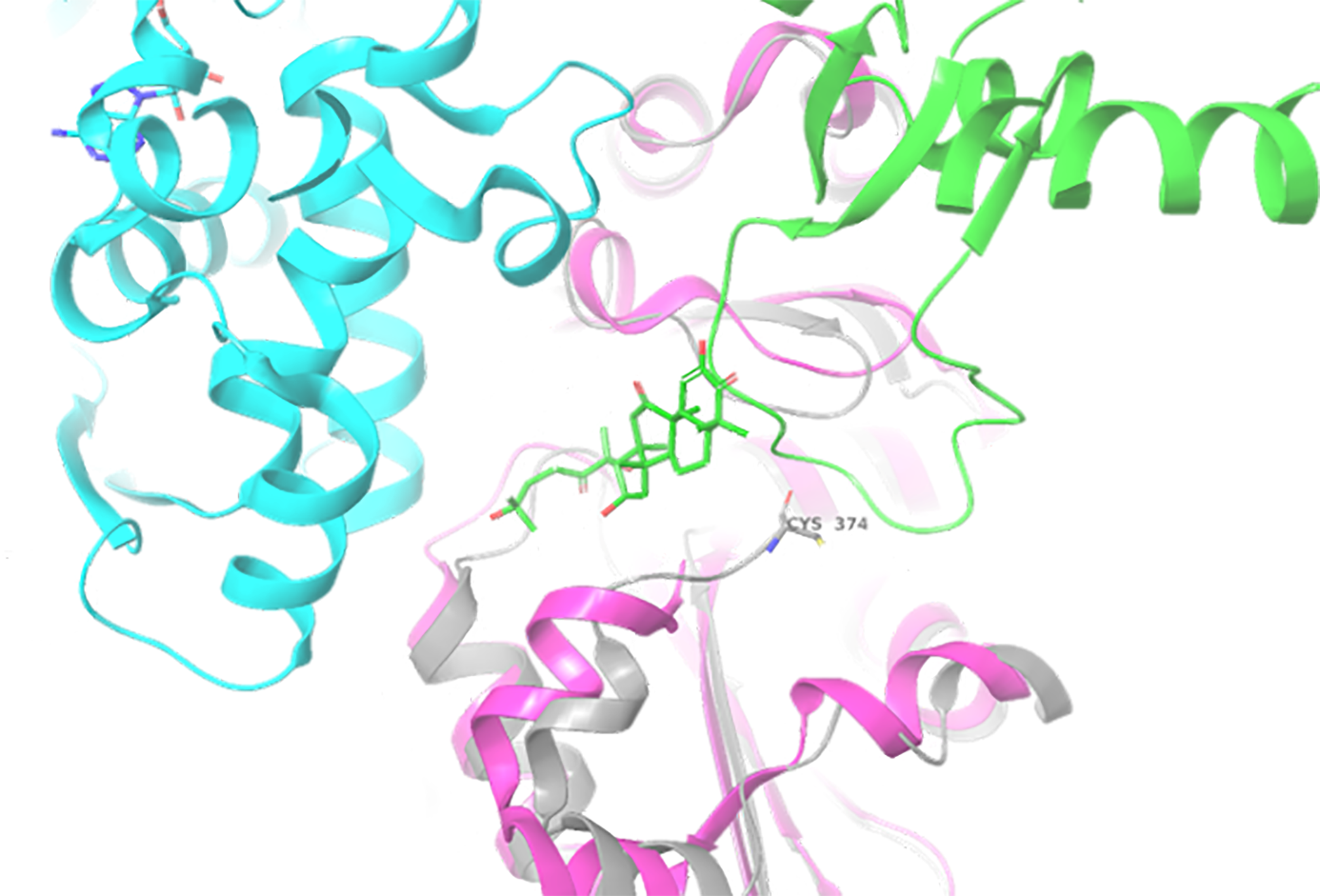


**Figure S.2.** G-actin monomer intersection on the F-actin structure.
